# The potential clinical utility of cell-free DNA for gastric cancer patients treated with nivolumab monotherapy

**DOI:** 10.1038/s41598-023-32645-x

**Published:** 2023-04-06

**Authors:** Chiaki Inagaki, Hisato Kawakami, Daichi Maeda, Daisuke Sakai, Shinya Urakawa, Kentaro Nishida, Toshihiro Kudo, Yuichiro Doki, Hidetoshi Eguchi, Hisashi Wada, Taroh Satoh

**Affiliations:** 1 Department of Frontier Science for Cancer and Chemotherapy, Graduate School of Medicine, Osaka University, Suita, 565-0871 Japan; 2 Department of Medical Oncology, Kindai University Faculty of Medicine, 377-2 Ohno-higashi, Osaka-sayama, Osaka 589-8511 Japan; 3 Department of Molecular and Cellular Pathology, Graduate School of Medicine, Kanazawa University, Kanazawa, 920-1192 Japan; 4 Center for Cancer Genomics and Personalized Medicine, Osaka University Hospital, Suita, 565-0871 Japan; 5 Department of Clinical Research in Tumor Immunology, Graduate School of Medicine, Osaka University, Suita, 565-0871 Japan; 6 Department of Medical Oncology, Osaka International Cancer Institute, Osaka, 541‐8567 Japan; 7 Department of Gastroenterological Surgery, Graduate School of Medicine, Osaka University, Suita, 565-0871 Japan

**Keywords:** Gastric cancer, Cancer, Predictive markers

## Abstract

To assess the potential clinical utility of cell-free DNA (cfDNA)-based biomarkers for identifying gastric cancer (GC) patients who benefit from nivolumab. From 31 GC patients treated with nivolumab monotherapy (240 mg/body, Bi-weekly) in 3rd or later line setting, we prospectively collected blood samples at baseline and before the 3rd dose. We compared cfDNA-based molecular findings, including microsatellite instability (MSI) status, to tissue-based biomarkers. We assessed the clinical value of blood tumor mutation burden (bTMB) and copy number alterations (CNA) as well as the cfDNA dynamics. The concordance between deficient-MMR and cfDNA-based MSI-high was 100% (3/3). Patients with bTMB ≥ 6 mut/Mb had significantly better progression-free survival (PFS) and overall survival (OS); however, such significance disappeared when excluding MSI-High cases. The combination of bTMB and CNA positivity identified patients with survival benefit regardless of MSI status (both PFS and OS, *P* < 0.001), with the best survival in those with bTMB^≥6mut/Mb^ and CNA^negative^. Moreover, patients with decreased bTMB during treatment had a better disease control rate (*P* = 0.04) and longer PFS (*P* = 0.04). Our results suggest that a combination of bTMB and CNA may predict nivolumab efficacy for GC patients regardless of MSI status. bTMB dynamics have a potential utility as an on-treatment biomarker.

## Introduction

Gastric cancer is the fifth most common cancer in incidence and the fourth leading cause of cancer mortality worldwide^1^. While cytotoxic chemotherapy is the mainstay of treatment for recurrent or advanced disease, the emergence of immune checkpoint inhibitors (ICIs), such as anti-programmed cell death-1 (PD-1) or programmed cell death ligand-1 (PD-L1) monoclonal antibodies, has changed the treatment landscape and treatment strategy^2–7^.

A PD-1 antibody, nivolumab, a fully human immunoglobulin (Ig) G4 monoclonal antibody targeting PD-1, has been approved for the third line and later treatment for advanced gastric cancer patients, based on the results of a phase III randomized clinical trial, ATTRACTION-2^2^, which. In this study, nivolumab showed significant benefit in overall survival (OS) compared to placebo (median OS, 5.26 months vs. 4.14 months, hazard ratio [HR] 0.63, *P* < 0.001). Although the primary endpoint of survival was achieved, the objective response rate (ORR) was only 11.2%, with a disease control rate (DCR) of 40.3%. There has been great interest in developing biomarkers that predict the response of ICIs and enable the selection of suitable gastric cancer patients to benefit from ICIs^3,8^.

Currently, PD-L1 expression is the most studied biomarker for ICI, which is used to stratify patients who obtain benefit from ICI monotherapy in the first line setting for non-small cell lung cancer and head and neck squamous cell cancer^9,10^. Although some research has been conducted to find an optimal PD-L1 cut-off value for gastric cancer, evidence suggests limited usefulness for PD-L1 expression status in this disease^2–7,11–14^. For gastric cancer, the most well-established biomarker for ICIs has been mismatch repair (MMR) status^3,8,15,16^. Another candidate biomarker includes Epstein–Barr virus (EBV) positivity^15,17^, which is present in approximately ~ 30% of gastric cancers^3^. EBV-positive tumors tend to have elevated PD-L1/2 expression and upregulation of immune mediated signaling^18^. These findings suggest the favorable antitumor efficacy of ICI for this subgroup; however, results have been inconsistent^12,15^. Tumor mutation burden (TMB), which is considered to be a marker for increased immunogenic neoantigens, has also been studied as a predictor of response to immunotherapy, including gastric cancer. Tissue TMB (tTMB) high status, which is defined as ≥ 10 mut/Mb using FoundationOne CDx as a companion diagnostic assay, has a broad indication for pembrolizumab monotherapy^19^. The usefulness of TMB in predicting the treatment response of ICIs for gastric cancer patients has been evaluated in several studies with various conclusions, though biomarker analysis of clinical trials using ICIs in gastric cancer revealed patients with higher TMB value had more favorable clinical efficacy from ICIs^12,15,20,21^.

While most of these established biomarkers require tumor tissue for evaluation, there is difficulty in evaluation due to a high degree of intratumor and inter-tumor heterogeneity that exists in gastric cancer^8,13,14^, which may hinder advancing translational research. Moreover, patients often face safety concerns when undergoing repeated tumor biopsies that may be needed to obtain sufficient tissue for biomarker analysis^3,8^.

Liquid biopsy is a rapidly growing technology that detects molecular profiling of cancer by analyzing circulating tumor cells (CTCs), cell-free DNA (cfDNA), exosomes, etc., which are isolated from blood^22^. This blood-based technology is minimally invasive and may be an alternative to tissue biopsy in advancing biomarker research^22^. Also, it provides a more convenient option than tissue re-biopsy for assessing real-time dynamics of biomarker status in cancer patients. Therefore, we conducted this analysis to investigate the potential of liquid biopsy to predict the efficacy of nivolumab monotherapy for gastric cancer patients using a next-generation sequencing (NGS)-based assay that analyzes cfDNA. We evaluated liquid-based molecular characteristics at baseline (pre-treatment) and their dynamics by analyzing paired blood samples (pre-treatment and on-treatment). We investigated the association between liquid-based molecular characteristics and tissue-based immunohistochemistry (IHC) results. We also explore the association between molecular characteristics and the efficacy of nivolumab in patients with advanced gastric cancer.

## Results

### Patients’ characteristics and molecular landscape

**Figure 1:**
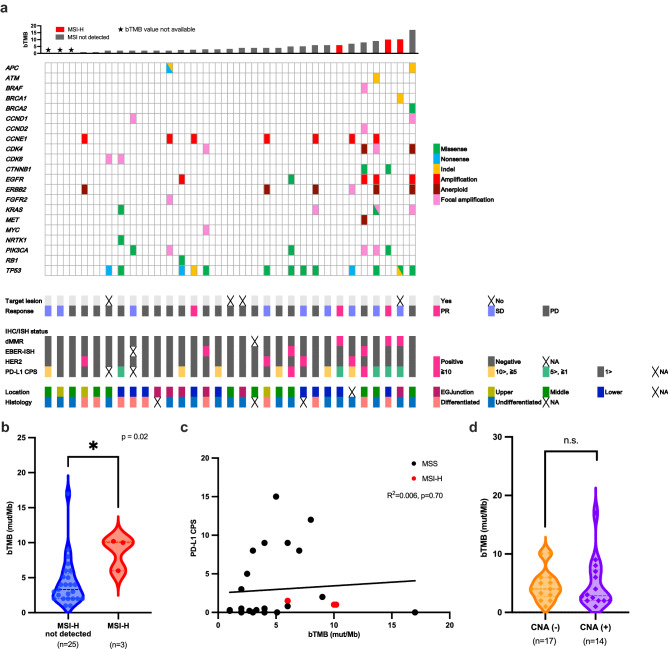
The association between the clinicopathological and genomic features of the study population. (**a**) The landscape of pathological feature observed in tissue samples and genomic alterations detected in baseline plasma samples of 31 gastric cancer patients. (**b**) Comparison of bTMB distribution between tumors with versus without MSI-H. (**c**) Relationship between bTMB and PD-L1 CPS. (**d**) Comparison of bTMB distribution between tumors with or without CNA. *bTMB* blood tumor mutation burden, *CNA* copy number alteration, *dMMR* mismatch repair-deficient, *EBER-ISH* Epstein–Barr virus (EBV)-encoded RNA in-situ hybridization, *HER2* human epidermal growth factor receptor 2, *IHC* immunohistochemistry, *ISH* in-situ hybridization, *MSI-H* microsatellite instability-high, *PD-L1 CPS* programmed death ligand 1 combined positive score, *PD* progressive disease, *PR* partial response, *SD* stable disease.

A total of 32 patients whose baseline blood samples (just before initiation of nivolumab treatment) were enrolled in this study, and the result of next-generation sequencing were analyzed for 31 patients. Among them, paired blood samples, including baseline and collection before the third treatment administration, were obtained from 20 patients (Supplemental Fig. 1b). Patient characteristics are shown in Table 1. Tissue HER2 status was available for all 31 patients. MMR, and EBV status were evaluated using archival samples in 30 patients. dMMR, HER2-positive, and EBV-positive were identified in 3 (9.7%), 4 (12.9%), and 3 (9.7%) patients, respectively, without overlap (Fig. 1a, Supplemental Fig. 2a). Of 29 patients with samples evaluable for PD-L1 status, PD-L1 CPS ≥ 1 was found in 13 patients (44.8%) and PD-L1 CPS ≥ 5 was observed in 8 patients (27.6%). All three dMMR tumors had PD-L1 CPS 1 ≤ to < 5 (Fig. 1a, and Supplemental Fig. 2a). One of four HER2-positive patients had PD-L1 CPS ≥ 5 whereas three others were PD-L1 negative. Two of three EBV-positive patients were PD-L1 CPS ≥ 10. There were no significant differences in the median value of PD-L1 CPS between EBV positive and negative patients (median, 12 vs. 0.5, *P* = 0.27; Supplemental Fig. 2b).

**Table 1:** Baseline characteristics.

**Number of patients (n)**	**31**
Median age, years (median, range)	64 (31–77)
Male/female (n, %)	22 (71.0)/9 (29.0)
ECOG PS 0/1 (n, %)	13 (41.9)/18 (58.1)
Primary tumor location (n, %)	
Esophagogastric junction	6 (19.4)
Upper	3 (9.7)
Middle	11(35.5)
Lower	10 (32.3)
Unknown	1 (3.2)
No. of metastatic site (median, range)	2 (1–4)
Metastatic site (n, %)	
Liver	15 (48.4)
Lung	4 (12.9)
Lymph node	20 (64.5)
Peritoneum	13 (41.9)
Histology (n, %)	
Differentiated	10 (32.3)
Undifferentiated	18 (58.1)
Unknown	3 (9.7)
HER2 status (n, %)	
Positive	4 (12.9)
Negative	27 (8.7)
EBER-ISH (n, %)	
Positive	3 (9.7)
Negative	27 (12.9)
NA	1 (3.2)
MMR status (n, %)	
dMMR	3 (9.7)
pMMR	27 (8.7)
NA	1 (3.2)
PD-L1 CPS (n, %)	
≥ 5	8 (25.8)
> 5, ≥ 1	5 (16.1)
1 >	16 (51.6)
NA	2 (6.5)
No. of previous regimen (median, range)	3 (2–7)

*dMMR* mismatch repair-deficient, *EBER-ISH* Epstein–Barr virus (EBV)-encoded RNA in-situ hybridization, *ECOG PS* Eastern Cooperative Oncology Group performance status, *HER2* human epidermal growth factor receptor 2, *MMR* mismatch repair, *NA* not available, *PD-L1 CPS* programmed death ligand 1 combined positive score, *pMMR* mismatch repair-proficient.

The result of next-generation sequencing of baseline plasma samples from 31 patients were analyzed, which revealed that 19 had at least one pathogenic and/or likely pathogenetic gene alteration (19/31, 61.3%, Fig. 1a). Among these 19 patients, a total of 30 gene mutations were found in 17 patients, whereas a total of 36 copy number alterations (CNAs) were found in 14 patients. The most frequently altered gene was *TP53* (13/31, 41.9%), followed by *CCNE1* (7/31, 22.6%), and *ERBB2* (6/31, 19.4%). Among four HER2-positive tumors, three had *ERBB2* CNA (3/4,75%, Supplemental Table 1a). Two of the three EBV-positive tumors had *PIK3CA* alteration, and there was no statistical difference in the frequency of *PIK3CA* alteration between EBV positive and negative tumors (2/3 [66.7%] vs. 4/27 [14.8%], *P* = 0.09, Supplemental Table 1b). Plasma-based MSI status was successfully evaluated on all baseline samples, with complete correlation to tissue analysis (3 dMMR/MSI-H, 27 MMR-proficient (pMMR)/MSI-H not detected; Fig. 1a, Supplemental Table 1c). Additionally, no samples with MSI-H had CNA. bTMB was available for 28 patients. bTMB values ranged from 1 to 17 mut/Mb (Fig. 1a), with 50th (median) and 75th percentiles being 4 mut/Mb and 6 mut/Mb, respectively. bTMB was significantly higher in samples with versus without MSI-H (Fig. 1b), and all samples with MSI-H had bTMB of 6 mut/Mb or higher. The analysis of distribution between bTMB and CPS indicated no significant association (R^2^ = 0.006, *P* = 0.71; Fig. 1c). There was no significant difference in bTMB between plasma samples with or without CNA (*P* = 0.58, Fig. 1d).

### Association between immunotherapy efficacy and the result of IHC/ISH analysis

**Figure 2:**
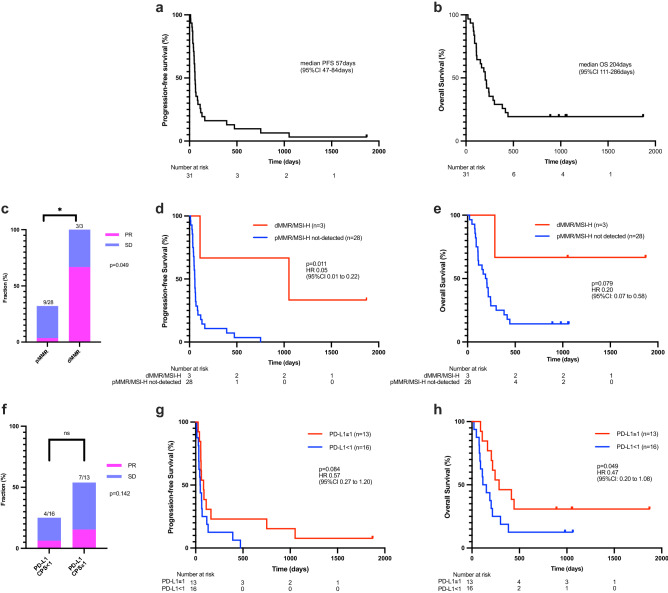
The clinical outcome of gastric cancer patients who received nivolumab monotherapy in the overall population, and its association with tissue-based biomarkers including dMMR and PD-L1 CPS. Kaplan–Meier estimates of progression-free survival (PFS, **a**) and overall survival (OS, **b**) of overall gastric cancer patients treated with nivolumab monotherapy. Disease control rate (DCR, **c**), and Kaplan–Meier estimates of PFS (**d**), as well as OS (**e**), according to dMMR status. Disease control rate (DCR,** f**), and Kaplan–Meier estimates of PSF (**g**), as well as OS (**h**), of patients stratified by PD-L1 CPS of 1. *dMMR* mismatch repair-deficient, *MSI-H* microsatellite instability-high, *MSS* microsatellite stable, *PD-L1 CPS* programmed death ligand 1 combined positive score, *PR* partial response, *SD* stable disease.

Among the 31 patients included in this analysis, nivolumab monotherapy conferred a median PFS and OS of 57 days [95% CI 47–84 days] and 204 days [95% CI 111–286 days], respectively (Fig. 2a,b). Three patients (12.9%) had a partial response (PR) and 5 patients had stable disease (SD) without pseudoprogression.

Among three dMMR/MSI-H cases, we found two PR and one SD, yielding a DCR of 100% (Fig. 2c), which was significantly higher (*P* = 0.049) than for cases that were pMMR and/or MSI-H not detected (DCR = 32.1%). PFS was also significantly longer in patients with dMMR/MSI-H compared to those with pMMR and/or MSI-H not detected (median PFS, 1052 days vs. 56 days, HR 0.05 [95% CI 0.01–0.22], *P* = 0.011; Fig. 2d). Patients with dMMR tumors also showed a non-significant trend toward improved OS (median OS, not reached (NR) vs. 195.5 days, HR 0.20 [95% CI 0.07–0.58], *P* = 0.079; Fig. 2e).

For PD-L1 CPS, there was no significant difference in DCR between patients with PD-L1 CPS ≥ 1 tumor vs. those with PD-L1 CPS < 1 tumor (7/13 [53.8%] vs. 4/16 [25.0%], *P* = 0.142; Fig. 2f). Although significant benefit in PFS was not observed (median PFS, 84 days vs. 48 days, HR 0.57 [95% CI 0.27–1.20], *P* = 0.083), patients with CPS ≥ 1 tumor had a significantly longer OS (median OS, 286 days vs. 133.5 days, HR 0.47 [95% CI 0.20–1.08], *P* = 0.049) compared to those with CPS < 1 tumor (Fig. 2g,h). We also analyzed patients’ survival using the cut-off value of CPS 5 but found no significant difference in either PFS or OS for those with CPS scores above or below 5 (median PFS, 58.5 days vs. 63 days, HR 1.08 [95% CI 0.47–2.45], *P* = 0.83; median OS, 210.5 days vs. 203.0 days, HR 0.96 [95% CI 0.38–2.44], *P* = 0.77; Supplemental Fig. 3a,b). No trends of HR enhancement in PFS or OS were observed with increasing CPS values (Supplemental Fig. 3c–f). For EBV, none of the positive cases (n = 3) responded to nivolumab. There was no significant difference in PFS and OS between EBV positive and negative patients (median PFS, 55.0 days vs. 58.5 days, HR 1.06 [95% CI 0.32–3.50], *P* = 0.37; median OS, 217.0 days vs. 195.5 days, HR 0.90 [95% CI 0.27–3.00], *P* = 0.77; Supplemental Fig. 4a,b). Among four HER2-positive cases, three had SD and one had PD. There was no significant survival benefit in HER2 positive vs. negative patients (median PFS, 147.0 days vs. 54.5 days, HR 0.37 [95% CI 0.13–1.07], *P* = 0.30; median OS, 252.0 days vs. 195.5 days, HR 0.78 [95% CI 0.23–2.59], *P* = 0.60; Supplemental Fig. 4c,d).

### Association between immunotherapy efficacy and baseline molecular landscape by liquid biopsy

We then examined the association between patients’ survival and the presence of specific genomic alterations detected in more than 10% of samples, including *TP53*, *CCNE1*, *CDK4*, *EGFR*, *ERBB2*, *KRAS*, and *PIK3CA*. However, the presence or absence of individual alterations was not associated with survival (Supplemental Fig. 5). Similarly, there was no significant survival difference between patients harboring tumors with or without CNA (median PFS, 60.0 days vs. 55.0 days, HR 0.92 [95% CI 0.45–1.88], *P* = 0.66; median OS, 203.5 days vs. 216.0 days, HR 1.06 [95% CI 0.48–2.33], *P* = 0.56; Supplemental Fig. 5).

**Figure 3:**
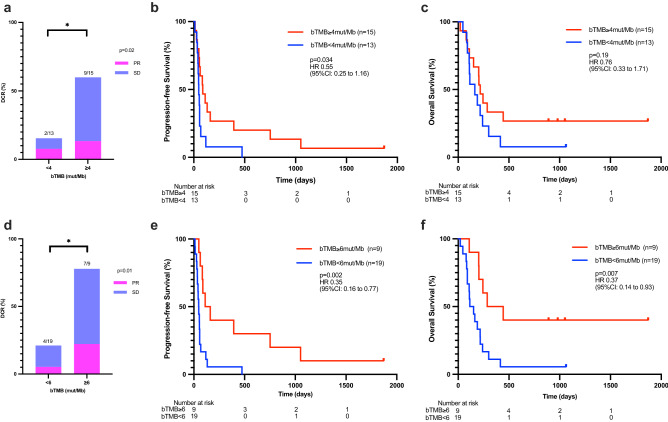
The association of blood tumor mutation burden (bTMB) with clinical outcome of gastric cancer patients received nivolumab monotherapy. Disease control rate (DCR,** a**), and Kaplan–Meier estimates of progression-free survival (PFS, **b**), as well as overall survival (OS, **c**) of patients stratified by bTMB of 4 mut/Mb. Disease control rate (DCR, **d**), and Kaplan–Meier estimates of PFS (**e**), as well as OS (**f**), of patients stratified by bTMB of 6 mut/Mb.

We next investigated whether bTMB is associated with nivolumab efficacy. We assessed tumor shrinkage and survival in groups stratified by 50th (median, bTMB = 4 mut/Mb) and 75th percentiles (bTMB = 6 mut/Mb) of bTMB (Fig. 3). Using the median cut-off value, DCR was significantly greater in patients with higher bTMB (9/15 [15.4%] vs. 2/13 [60.0%], *P* = 0.02), as was PFS (median PFS, 88 days vs. 48 days, HR 0.55 [95% CI 0.25–1.16], *P* = 0.034) but not OS (median OS 217 days vs. 164 days, HR 0.76 [95% CI 0.33–1.71], *P* = 0.19). However, using a bTMB cut-off of 6 mut/Mb (Fig. 3a–c), nivolumab clinical outcomes were improved with higher vs lower bTMB values. DCR was significantly greater (7/9 [77.7%] vs. 4/19 [21.1], *P* = 0.01), and both PFS (median PFS, 137 days vs. 47.5 days, HR 0.35 [95% CI 0.16–0.77], *P* = 0.002) and OS (median OS, 365 days vs. 133.5 days, HR 0.37 [95% CI 0.14–0.93], *P* = 0.007) were significantly longer in patients harboring tumor with bTMB ≥ 6 (Fig. 3). Given that MSI-H had higher bTMB (Fig. 1b), we examined the association between bTMB and survival in cases where MSI-H was not detected. We found a significant difference in PFS for higher bTMB values, using either a cut-off of 4 mut/Mb (median PFS, 86.0 days vs. 47.0 days, HR 0.55 [95% CI 0.24–1.27], *P* = 0.040) or 6 mut/Mb (median PFS, 88.0 days vs. 47.5 days, HR 0.54 [95% CI 0.23–1.29], *P* = 0.027; Supplemental Fig. 6a,b). On the other hand, a significant OS advantage was not observed for tumors with MSI-H not detected, regardless of bTMB cut-off value (bTMB ≥ 4 mut/Mb, median 230.5 days vs. 115.0 days, HR 0.50 [95% CI 0.20–1.28] *P* = 0.051; bTMB ≥ 6 mut/Mb, median 244.0 days vs. 133.5 days, HR 0.55 [95% CI 0.20–1.48], *P* = 0.053; Supplemental Fig. 6c,d).

**Figure 4:**
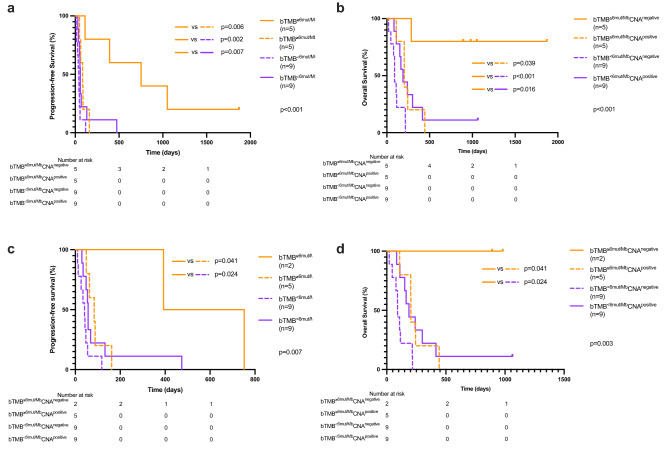
The association of bTMB and CNA combination with clinical outcome of gastric cancer patients treated with nivolumab monotherapy. Kaplan–Meier estimates of progression-free survival (PFS, **a**), as well as overall survival (OS, **b**) of patients stratified by the combination of bTMB and CNA in the overall population. Kaplan–Meier estimates of PFS (**c**), as well as OS (**d**) of patients stratified by the combination of bTMB and CNA when MSI-H cased excluded. *bTMB* blood tumor mutation burden, *CNA* copy number alteration, *MSI-H* microsatellite instability high.

Given that the presence of CNA may result in a state of immune evasion^23,24^, we asked whether survival outcomes were affected by bTMB in combination with presence or absence of CNA. Significant differences between groups were observed for PFS and OS. (bTMB^≥6mut/Mb^/CNA^negative^ vs. bTMB^≥6mut/Mb^/CNA^positive^ vs. bTMB^<6mut/Mb^/CNA^negative^ vs. bTMB^<6mut/Mb^/CNA^positive^; median PFS, 752 days vs. 84 days vs. 37.5 vs. 55.5 days, *P* < 0.001; median OS, not reached vs. 204 days vs. 99.5 days vs. 176 days, *P* < 0.001; Fig. 4a,b). In post-hoc analyses, we found that patients harboring tumors classified as bTMB^≥6mut/Mb^/CNA^negative^ had significantly longer PFS and OS than those in the other three categories: bTMB^≥6mut/Mb^/CNA^positive^ (median PFS, *P* = 0.006; median OS, *P* = 0.039), bTMB^<6mut/Mb^/CNA^negative^ (median PFS, *P* = 0.002; median OS, *P* < 0.001), and bTMB^<6mut/Mb^/CNA^positive^ (median PFS, *P* = 0.007; median OS *P* = 0.016). This effect persisted after excluding the MSI-H tumors, all of which were bTMB^≥6mut/Mb^/CNA^negative^ (median PFS, bTMB^≥6mut/Mb^/CNA^negative^ vs. bTMB^≥6mut/Mb^/CNA^positive^ vs. bTMB^<6mut/Mb^/CNA^negative^ vs. bTMB^<6mut/Mb^/CNA^positive^, 572.5 days vs. 84 days vs. 37.5 days vs. 55.5 days, *P* = 0.007). For OS, the median values for each respective group were not reached vs. 204 days vs. 99.5 days, vs. 176 days, *P* = 0.003 (Fig. 4c,d). We further found significant difference both in PFS and OS for patients with bTMB^≥6mut/Mb^ CNA^negative^ compared to patients with bTMB^≥6mut/Mb^ CNA^positive^ (median PFS, *P* = 0.041; median OS *P* = 0.041), TMB^<6mut/Mb^ CNA^negative^ (median PFS, *P* = 0.024; median OS, *P* = 0.024). These findings suggest that, when using a cfDNA panel with a relatively small genomic footprint, bTMB in combination with CNA information may predict clinical outcomes for gastric cancer patients treated with nivolumab monotherapy.

### Association between immunotherapy efficacy and liquid biopsy dynamics

Using pre- and on- treatment samples, we evaluated the dynamics of genomic alterations detected in liquid biopsy as a potential predictor for nivolumab efficacy. Among 20 paired samples, 19 were available for evaluation of maxVAF value and bTMB at both timepoints (Supplemental Fig. 1b).

We examined the association between maxVAF change and treatment response. Ten patients had decreased ΔmaxVAF (pre-treatment maxVAF minus on-treatment maxVAF), whereas 9 patients had non-decreased ΔmaxVAF (Supplemental Fig. 7a). We found no significant association between the maxVAF change (decreased vs. not decreased) and tumor response (R^2^ = 0.01, *P* = 0.70; Supplemental Fig. 7b). DCR was not significantly different in patients with decreased ΔmaxVAF compared to those with non-decreased ΔmaxVAF (5/7 [71.4%] vs. 3/12 [25.0%], *P* = 0.07; Supplemental Fig. 7c). Similarly, PFS and OS were not significantly different in patients with decreased ΔmaxVAF compared to those with non-decreased ΔmaxVAF (median PFS, 88 days vs. 56 days, HR 0.64 [95% CI 0.24–1.70], *P* = 0.24; median OS, 286 days vs. 202 days, HR 0.71 [95% CI 0.25–2.03], *P* = 0.20; Supplemental Fig. 7d,e).

**Figure 5:**
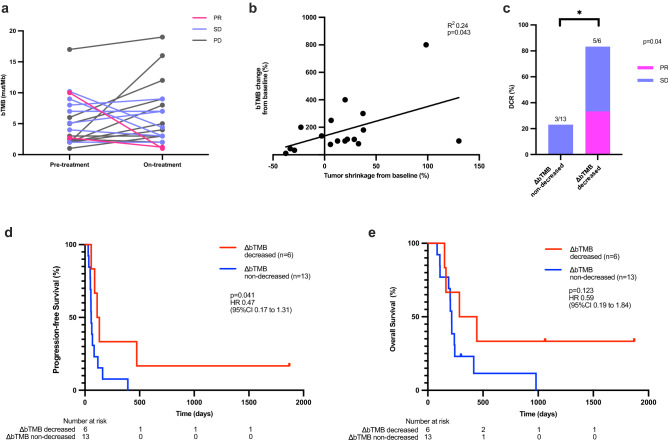
The association between ΔbTMB dynamics and clinical outcome of gastric cancer patients treated with nivolumab monotherapy. (**a**) Association between tumor response and changes of bTMB between pre- vs. on-treatment (immediately before the third cycle) samples. (**b**) Correlation between bTMB change from the baseline and degree of tumor shrinkage. (**c**) Comparison of disease control rate (DCR) between cases with decreased bTMB vs. non-decreased bTMB. Kaplan–Meier estimates of progression-free survival (PFS, **d**) and overall survival (OS, **e**) of patients with decreased- vs. non-decreased- bTMB. *bTMB* blood tumor mutation burden.

We next focused on the dynamics of bTMB. Six patients had decreased ΔbTMB (pre-treatment bTMB minus on-treatment bTMB) and 13 patients had non-decreased ΔbTMB (Fig. 5a). Analysis of the association between dynamics of bTMB and treatment response revealed significant and weak association between the magnitude of bTMB change and tumor shrinkage (R^2^ = 0.24, *P* = 0.043; Fig. 5b). DCR was significantly higher in patients with decreased ΔbTMB compared to those with non-decreased ΔbTMB (5/6 [83.3%] vs. 3/13 [23.1%], *P* = 0.04; Fig. 5c). Moreover, we found that PR was observed solely in patients with decreased ΔbTMB (2/6, 33.3%). We further found that significantly longer PFS was observed in patients with decreased ΔbTMB compared to those with non-decreased ΔbTMB (median PFS, 122 days vs. 57 days, HR 0.47 [95% CI 0.17–1.31], *P* = 0.041; Fig. 5d), although OS in patients with decreased ΔbTMB was not significantly longer compared to those with non-decreased ΔbTMB (median OS, 365 days vs. 216 days, HR 0.59 [95% CI 0.19–1.84], *P* = 0.123; Fig. 5e). These findings suggest that ΔbTMB accurately reflects treatment response, raising a potential to serve as an early indicator of nivolumab treatment benefit.

## Discussion

The exploration of biomarkers to predict the therapeutic efficacy of immune checkpoint inhibitors in gastric cancer is ongoing. Well-recognized biomarkers for gastric cancer are tissue-based assays such as PD-L1, dMMR, and EBV status^3,8^, which may be difficult to assess in gastric cancer with insufficient tumor biopsy quantity or quality, especially in undifferentiated tumors and those with heterogeneity^8^. Blood-based assays have the potential to overcome such limitations and may be useful when applied clinically in gastric cancer.

In the current study, we first analyzed the utility of known tissue- and blood-based biomarkers in predicting nivolumab treatment efficacy. Patients with dMMR tumors, compared to those with pMMR tumors, demonstrated excellent survival, consistent with previous findings^15,17^. We found that the relationship between PD-L1 CPS and the efficacy of nivolumab was insignificant, as shown by various PD-L1 CPS cut-off values that did not produce consistent trends. Indeed, the exploratory analysis of ATTRACTION-2 concluded that PD-L1 tumor positive score (TPS) was not associated with survival benefit of nivolumab monotherapy in the later line setting for gastric cancer^2^, while KEYNOTE-061 and KEYNOTE-062 demonstrated the promising clinical utility of PD-L1 CPS in defining gastric cancer patients benefit more from pembrolizumab monotherapy in an earlier clinical setting^5,6^. Considering that most of the tissue samples examined in our study were obtained at the time of diagnosis, they may not reflect the status of the tumor just prior to treatment. Therefore, the predictive value of tumor PD-L1 status, either CPS or TPS, for ICI efficacy may be dependent on when tissue is collected. Also, we did not find clinical benefits of nivolumab in EBV-positive patients. These differences between previous studies^2,4–7,12,15,17^ and ours, in the reliability of PD-L1 and EBV status as a biomarker of ICI efficacy, is possibly due to the relatively small number of patients in the current analysis.

Regarding molecular features on liquid biopsy, one of our important findings was that plasma-based MSI-H and tissue-based dMMR status were in 100% concordance. As shown in previous studies^16,25^, MSI-H determination by liquid biopsy may provide a convenient approach for determining ICI eligibility for gastric cancer patients. On the other hand, we did not observe any single genetic alteration that was associated with a therapeutic effect of nivolumab. We, therefore, explored the broader mutational landscape in each plasma sample by investigating the impact of bTMB on the prediction of nivolumab efficacy. As cut-off values for bTMB by the version of the assay used in this study have not been established, we examined the cut-off values of 4 mut/Mb and 6 mut/Mb of bTMB, which are the 50th and 75th percentile for bTMB, respectively. We found that patients with bTMB ≥ 6 mut/Mb had longer PFS and OS compared to patients with bTMB < 6 mut/Mb. Data have suggested a lower range of bTMB is mainly distributed in MSS gastric cancer patients^26,27^, while high bTMB is often observed in MSI-H gastric tumors^27,28^. We thus excluded MSI-H cases from the analysis and found that the significant improvement in OS was lost, consistent with a post-hoc analysis of a clinical trial in which tissue-based TMB was assessed^21^. Despite the limited number of cases in our study, these results suggest that there may be some predictive value for high bTMB alone for gastric cancer patients treated with nivolumab^28^.

Given that previous studies have shown the usefulness of the combination of TMB and CNA obtained by tissue-based whole exome sequence (WES) and NGS panel assay in the prediction of ICI efficacy^29,30^, we integrated bTMB and blood-based CNA and analyzed its association with treatment outcome. Here, we first demonstrated that patients with bTMB^≥6mut/Mb^CNA^negative^ had the best benefit from nivolumab. CNA, one of the hallmarks of cancer, is known to result in immune evasion^31^. CNA high tumors were shown to have an immune evading environment compared to CNA low tumors due to upregulated expression of cell cycle markers and decreased expression of cytotoxic markers and immune cell infiltration, thus leading to a poor prognosis when treated with ICI^23,24^. In general, elevated tissue TMB has been shown to be associated with increased CD8 T-cell infiltration; however, the positive correlation between TMB and CD8 T-cell infiltration is not observed in gastric cancer with CNA high tumors^24,32^. Given that these findings are based on the tissue-based assay, we wished to determine whether they could be applied to a blood-based relatively small NGS panel assay as well^33^. Moreover, we did not evaluate immune cell infiltration in tumor tissue. Further study is therefore needed in this regard. Nonetheless, these data support our finding of the usefulness of the bTMB and CNA combination in the prediction of nivolumab in this setting, which warrants confirmation in a larger cohort.

In our analysis of the association between nivolumab efficacy and liquid biopsy dynamics, including two parameters, ΔmaxVAF and ΔbTMB. We found a significant PFS improvement in patients with decreased ΔbTMB but not in patients with ΔmaxVAF, suggesting a potential utility of ΔbTMB as an on-treatment biomarker for PFS. Dynamics of bTMB, as well as maxVAF, have been found to be correlated with treatment response and prolonged survival^34–40^, given that both bTMB and maxVAF innately reflect tumor burden^39,41^; however, there are few studies comparing the utility of maxVAF and bTMB dynamics for predicting the treatment efficacy of ICI^42^. A recent study demonstrated correlation with outcomes for cancer patients treated with ICI, including those with gastrointestinal cancers, was observed for maxVAF changes, but not for bTMB changes^42^. The results of that study, however, are inconsistent with our results. Possible reasons include a different ctDNA assay platform and schedules for blood sample collection between the studies (Supplementary Table 2). Furthermore, that study and ours were both conducted with a small number of patients (n = 84 vs. n = 20). Given that there are no standard methods to quantitate ctDNA for maxVAF and bTMB calculation and those to evaluate their association with clinical outcomes at the moment^43^, further study and harmonization are strongly needed in order to use liquid biopsy efficiently in a clinical practice setting^43^.

The current study has several limitations. First, this study included a small sample size, which limits our ability to draw definitive conclusions from the exploratory analyses that we conducted. Nevertheless, our findings may inform larger studies that are better powered to confirm our hypotheses. Second, the plasma-based NGS assay that we used in this study, with a genomic footprint of approximately 150 kb, is much smaller than that of commercially available tissue-based NGS assays which report TMB^33,44,45^. This might affect the determination of bTMB and CNA status in gastric cancer patients. Additionally, considering the potential impact of tumor burden and the amount of tumor shedding, bTMB may not be as reliable and stable as tTMB^46^. A cautious interpretation of our result is therefore warranted. Finally, due to the nature of the observational study, we did not strictly set the assessment schedule of the CT scan, which may have missed the short-term immune response.

In conclusion, we demonstrated that, although bTMB based on an assay with a small genomic footprint has limited utility as a predictive biomarker for nivolumab treatment of advanced gastric cancer, a combination of bTMB and CNA status may have the potential to predict the efficacy of nivolumab monotherapy in the 3rd or later line setting Our analysis also raises the clinical utility of ΔbTMB in the monitoring of nivolumab monotherapy in this setting as an on-treatment biomarker. Further prospectively planned analyses with a larger cohort are needed to confirm these findings and to use liquid biopsy more efficiently in the real-world practice for gastric cancer patients treated with ICIs.

## Materials and methods

### Patients and sample collection

Patients included in this study were those who (i) consented to the collection of their blood for research purposes and (ii) were treated with nivolumab (240 mg, every 2 weeks) for advanced gastric cancer at Osaka University Hospital from December 2014 to May 2018. Blood samples were collected at baseline (just before initiation of nivolumab treatment) and immediately before the third administration (6 weeks after the first dose, Supplemental Fig. 1b). This study was approved by the Osaka University ethical committee (No. 764, No. 765) in accordance with the Declaration of Helsinki.

### Plasma samples analysis

Plasma samples were isolated via centrifugation and stored at − 80 °C until cfDNA analysis. For cfDNA analysis, next-generation sequencing was performed using Guardant360, as previously described^47^. This assay detects SNVs, indels, fusions, and copy number alterations in 74 genes (Supplementary Table 3) with a reportable range of ≥ 0.04, ≥ 0.02, ≥ 0.04% and ≥ 2.12 copies, respectively, as well as microsatellite instability-high (MSI-H). 3–5 ml of frozen plasma samples were shipped to the central laboratory, which is CLIA-certified, CAP-accredited, and New York State Department of Health-approved. After extraction from plasma, 5–30 ng of cfDNA was labeled with nonredundant oligonucleotides (molecular barcoding), enriched using targeted hybridization capture, and sequenced on the Illumina NextSeq 550 platform. Base call files generated by Illumina’s RTA software v.2.12 were demultiplexed using bcl2fastq v.2.19 and processed as previously described^47^. The processed reads were then aligned to hg19 using the Burrows–Wheeler aligner–MEM algorithm. Somatic cfDNA alterations were identified using a proprietary bioinformatics pipeline and ctDNA fraction was measured by the maximum variant allelic frequency (maxVAF).

The reported variants were filtered based on several sources, including OncoKB, Clin Var, and COSMIC, and selected variants of known or likely pathogenic status were identified and represented on an oncoprint. Plasma-based MSI assessment was conducted by sequencing microsatellite loci, as previously described^16^. The blood tumor mutation burden (bTMB) was determined by normalization to the mutation burden expected for the tumor type and ctDNA fraction, as derived from a training set of 10,543 consecutive clinical samples, and is reported as a bTMB score.

### Tissue immunohistochemical analysis

Archival formalin-fixed paraffin-embedded (FFPE) tumor specimens with the pathological report, which included HER2 status, were retrospectively collected for IHC analysis, if residual samples were available. IHC analysis, such as for PD-L1, MMR, and chromogenic in situ hybridization for EBV-encoded RNA (EBER-ISH) using fluorescein-labeled peptide nucleic acid probes (EBV PNA Probe/Fluorescein, Agilent), were performed on FFPE tumor samples and assessed by an established pathologist.

For PD-L1 evaluation, IHC staining was performed using PD-L1 IHC 28-8 pharmDx (Dako). The level of PD-L1 protein expression was determined using the combined positive score (CPS), which was calculated as the number of PD-L1-stained cells (tumor cells, lymphocytes, and macrophages) divided by the total number of viable tumor cells and multiplied by 100. Tumor PD-L1 positivity was defined as CPS ≥ 1%.

MMR status was determined by IHC for the following proteins; anti-mutL homolog 1 (MLH1; Clone ES05, Agilent Technologies), anti-mutS homolog 2 (MSH2; Clone FE11, Agilent Technologies), anti-postmeiotic segregation increased 2 (PMS2; Clone EP51, Agilent Technologies), and anti-mutS homolog 6 (MSH6; Clone EP49, Agilent Technologies), in FFPE samples. MMR-deficient (dMMR) was defined as a tumor that lacked staining for at least one of the MMR proteins.

### Outcomes and statistical analysis

Patients’ clinical data were retrospectively obtained from electronic records. We evaluated the ORR, DCR, progression-free survival (PFS), and OS. Tumor response was assessed in patients with evaluable lesions using the Response Evaluation Criteria in Solid Tumors version 1.1^48^. The ORR was defined as the proportion of patients with the best overall response of complete response (CR) or partial response (PR). The DCR was defined as the proportion of patients with CR, PR, or stable disease. Pseudoprogression was defined as 25% initial increase in tumor burden and subsequent imaging evaluations that fulfilled the criteria of partial response^49^. The PFS was defined as the interval from the start of treatment until disease progression or death from any cause or the last follow-up visit and estimated by the Kaplan–Meier method. Statistical analyses were performed using GraphPad and EZR^50^. Categorical and quantitative data were compared using the Fisher’s exact test and Mann–Whitney *U* test, respectively. Survival analysis was estimated by the Kaplan–Meier method with the log-rank test and presented as hazard ratios (HRs) with 95% confidence intervals (CIs). A two- sided *P* < 0.05 was considered significant. For multiple comparisons, if statistically significant existed among all groups, no post-hoc comparison adjustment was used.

### Ethical approved and consent to participate

This study was approved by the Osaka University ethical committee (No. 764, No. 765) in accordance with the Declaration of Helsinki. Written informed consent was obtained from all patients who were enrolled in this study.

## Supplementary Information

Supplementary Information.

## Data Availability

The datasets generated during and/or analyzed during the study are not publicly available due to restrictions of the research ethics protocol but are available from the corresponding author upon reasonable request.

## References

[1] Sung H (2021). Global Cancer Statistics 2020: GLOBOCAN estimates of incidence and mortality worldwide for 36 cancers in 185 countries. CA Cancer J. Clin..

[2] Kang YK (2017). Nivolumab in patients with advanced gastric or gastro-oesophageal junction cancer refractory to, or intolerant of, at least two previous chemotherapy regimens (ONO-4538-12, ATTRACTION-2): A randomised, double-blind, placebo-controlled, phase 3 trial. Lancet.

[3] Kang BW, Chau I (2020). Current status and future potential of predictive biomarkers for immune checkpoint inhibitors in gastric cancer. ESMO Open..

[4] Kang Y-K (2022). Nivolumab plus chemotherapy versus placebo plus chemotherapy in patients with HER2-negative, untreated, unresectable advanced or recurrent gastric or gastro-oesophageal junction cancer (ATTRACTION-4): A randomised, multicentre, double-blind, placebo-controlled, phase 3 trial. Lancet Oncol..

[5] Shitara K (2018). Pembrolizumab versus paclitaxel for previously treated, advanced gastric or gastro-oesophageal junction cancer (KEYNOTE-061): A randomised, open-label, controlled, phase 3 trial. Lancet.

[6] Shitara K (2020). Efficacy and safety of pembrolizumab or pembrolizumab plus chemotherapy vs chemotherapy alone for patients with first-line, advanced gastric cancer: The KEYNOTE-062 phase 3 randomized clinical trial. JAMA Oncol..

[7] Janjigian YY (2021). First-line nivolumab plus chemotherapy versus chemotherapy alone for advanced gastric, gastro-oesophageal junction, and oesophageal adenocarcinoma (CheckMate 649): A randomised, open-label, phase 3 trial. Lancet.

[8] Nakamura Y, Kawazoe A, Lordick F, Janjigian YY, Shitara K (2021). Biomarker-targeted therapies for advanced-stage gastric and gastro-oesophageal junction cancers: An emerging paradigm. Nat. Rev. Clin. Oncol..

[9] Reck M (2016). Pembrolizumab versus chemotherapy for PD-L1-positive non-small-cell lung cancer. N. Engl. J. Med..

[10] Burtness B (2019). Pembrolizumab alone or with chemotherapy versus cetuximab with chemotherapy for recurrent or metastatic squamous cell carcinoma of the head and neck (KEYNOTE-048): A randomised, open-label, phase 3 study. Lancet.

[11] Xie T (2021). Appropriate PD-L1 cutoff value for gastric cancer immunotherapy: A systematic review and meta-analysis. Front. Oncol..

[12] Wang F (2019). Safety, efficacy and tumor mutational burden as a biomarker of overall survival benefit in chemo-refractory gastric cancer treated with toripalimab, a PD-1 antibody in phase Ib/II clinical trial NCT02915432. Ann. Oncol..

[13] Zhou KI (2020). Spatial and temporal heterogeneity of PD-L1 expression and tumor mutational burden in gastroesophageal adenocarcinoma at baseline diagnosis and after chemotherapy. Clin. Cancer Res..

[14] Schoemig-Markiefka B (2021). Optimized PD-L1 scoring of gastric cancer. Gastric Cancer.

[15] Kim ST (2018). Comprehensive molecular characterization of clinical responses to PD-1 inhibition in metastatic gastric cancer. Nat. Med..

[16] Willis J (2019). Validation of microsatellite instability detection using a comprehensive plasma-based genotyping panel. Clin. Cancer Res..

[17] Mishima S (2019). Clinicopathological and molecular features of responders to nivolumab for patients with advanced gastric cancer. J. Immunother. Cancer.

[18] Bass AJ (2014). Comprehensive molecular characterization of gastric adenocarcinoma. Nature.

[19] Marabelle A (2020). Association of tumour mutational burden with outcomes in patients with advanced solid tumours treated with pembrolizumab: Prospective biomarker analysis of the multicohort, open-label, phase 2 KEYNOTE-158 study. Lancet Oncol..

[20] Lee KW (2022). Association of tumor mutational burden with efficacy of pembrolizumab ± chemotherapy as first-line therapy for gastric cancer in the phase III KEYNOTE-062 study. Clin. Cancer Res..

[21] Shitara K (2021). Molecular determinants of clinical outcomes with pembrolizumab versus paclitaxel in a randomized, open-label, phase III trial in patients with gastroesophageal adenocarcinoma. Ann. Oncol..

[22] Heitzer E, Haque IS, Roberts CES, Speicher MR (2019). Current and future perspectives of liquid biopsies in genomics-driven oncology. Nat. Rev. Genet..

[23] Davoli T, Uno H, Wooten EC, Elledge SJ (2017). Tumor aneuploidy correlates with markers of immune evasion and with reduced response to immunotherapy. Science.

[24] Derks S (2020). Characterizing diversity in the tumor-immune microenvironment of distinct subclasses of gastroesophageal adenocarcinomas. Ann. Oncol..

[25] Chakrabarti S (2022). Detection of microsatellite instability-high (MSI-H) by liquid biopsy predicts robust and durable response to immunotherapy in patients with pancreatic cancer. J. Immunother. Cancer..

[26] Saori M (2021). 80P Blood tumor mutational burden (bTMB) and efficacy of immune checkpoint inhibitors (ICIs) in advanced solid tumors: SCRUM-Japan MONSTAR-SCREEN. Ann. Oncol..

[27] Yoshino T (2021). Genomic immunotherapy (IO) biomarkers detected on comprehensive genomic profiling (CGP) of tissue and circulating tumor DNA (ctDNA). J. Clin. Oncol..

[28] Foote MB (2021). TMB cut-offs fail to predict benefit of PD-1 blockade in gastroesophageal adenocarcinoma in KEYNOTE-061. Ann. Oncol..

[29] Liu L (2019). Combination of TMB and CNA stratifies prognostic and predictive responses to immunotherapy across metastatic cancer. Clin. Cancer Res..

[30] Lu Z (2020). Tumor copy-number alterations predict response to immune-checkpoint-blockade in gastrointestinal cancer. J. Immunother. Cancer..

[31] Hanahan D (2022). Hallmarks of cancer: New dimensions. Cancer Discov..

[32] McGrail DJ (2021). High tumor mutation burden fails to predict immune checkpoint blockade response across all cancer types. Ann. Oncol..

[33] Fridland S (2021). Assessing tumor heterogeneity: Integrating tissue and circulating tumor DNA (ctDNA) analysis in the era of immuno-oncology—Blood TMB is not the same as tissue TMB. J. Immunother. Cancer..

[34] Jin Y (2020). The predicting role of circulating tumor DNA landscape in gastric cancer patients treated with immune checkpoint inhibitors. Mol. Cancer.

[35] Zhang Q (2020). Prognostic and predictive impact of circulating tumor DNA in patients with advanced cancers treated with immune checkpoint blockade. Cancer Discov..

[36] Zou W (2021). ctDNA predicts overall survival in patients with NSCLC treated with Pd-L1 blockade or with chemotherapy. JCO Precis. Oncol..

[37] Vega DM (2022). Changes in circulating tumor DNA reflect clinical benefit across multiple studies of patients with non–small-cell lung cancer treated with immune checkpoint inhibitors. JCO Precis. Oncol..

[38] Jiang T (2022). On-treatment blood TMB as predictors for camrelizumab plus chemotherapy in advanced lung squamous cell carcinoma: Biomarker analysis of a phase III trial. Mol. Cancer.

[39] Nabet BY (2020). Noninvasive early identification of therapeutic benefit from immune checkpoint inhibition. Cell.

[40] Nie W (2022). ctDNA-adjusted bTMB as a predictive biomarker for patients with NSCLC treated with PD-(L)1 inhibitors. BMC Med..

[41] Strijker M (2020). Circulating tumor DNA quantity is related to tumor volume and both predict survival in metastatic pancreatic ductal adenocarcinoma. Int. J. Cancer.

[42] Kato S (2022). Serial changes in liquid biopsy-derived variant allele frequency predict immune checkpoint inhibitor responsiveness in the pan-cancer setting. Oncoimmunology.

[43] Malla M, Loree JM, Kasi PM, Parikh AR (2022). Using circulating tumor DNA in colorectal cancer: Current and evolving practices. J. Clin. Oncol..

[44] Merino DM (2020). Establishing guidelines to harmonize tumor mutational burden (TMB): In silico assessment of variation in TMB quantification across diagnostic platforms: Phase I of the Friends of Cancer Research TMB Harmonization Project. J. Immunother. Cancer.

[45] Sha D (2020). Tumor mutational burden as a predictive biomarker in solid tumors. Cancer Discov..

[46] Schuurbiers M (2022). Biological and technical factors in the assessment of blood-based tumor mutational burden (bTMB) in patients with NSCLC. J. Immunother. Cancer.

[47] Odegaard JI (2018). Validation of a plasma-based comprehensive cancer genotyping assay utilizing orthogonal tissue- and plasma-based methodologies. Clin. Cancer Res..

[48] Eisenhauer EA (2009). New response evaluation criteria in solid tumours: Revised RECIST guideline (version 1.1). Eur. J. Cancer.

[49] Wolchok JD (2009). Guidelines for the evaluation of immune therapy activity in solid tumors: Immune-related response criteria. Clin. Cancer Res..

[50] Kanda Y (2013). Investigation of the freely available easy-to-use software ‘EZR’ for medical statistics. Bone Marrow Transplant..

