# Supplementary material for: The potential clinical utility of cell-free DNA for gastric cancer patients treated with nivolumab monotherapy
Source: Sci Rep. 2023 Apr 6;13:5652. doi: 10.1038/s41598-023-32645-x (PMC10079661; doi:10.1038/s41598-023-32645-x)

Supplemental Figure Legends

Supplemental Figure 1.

Study design. (a) Sample collection schedule. Blood sample collection was scheduled at baseline (just before initiation of nivolumab treatment) and immediately before the third administration (6 weeks after the first dose).

(b) CONSORT diagram of 31 patients enrolled and samples analyzed.

bTMB, blood tumor mutation burden, maxVAF; maximum variant allele frequency

Supplemental Figure 2

(a) Venn diagram shows the distribution of cases of dMMR, HER2-positive, EBV-positive, and PD-L1 CPS ≥1 or ≥5. b) Comparison of PD-L1 CPS between EBV-positive vs. negative cases. EBV, Epstein-Barr virus; dMMR, mismatch repair-deficient; HER2, human epidermal growth factor receptor 2; PD-L1 CPS, programmed death ligand 1 combined positive score;

Supplemental Figure 3

The clinical outcome of gastric cancer patients who received nivolumab monotherapy in the overall population, and its association with various PD-L1 CPS cut-offs. Kaplan–Meier estimates of progression-free survival (PFS, a) and overall survival (OS, b) of patients stratified by PD-L1 CPS ≥5 vs. <5. Kaplan–Meier estimates of PFS (c), and OS (d) of patients stratified by PD-L1 CPS ≥10 vs. <10. Hazard ratio and 95% confidence interval for PFS (e) and OS (f) in patient subgroups defined by PD-L1 CPS cut-off values of 1, 5, and 10. PD-L1 CPS, programmed death ligand 1 combined positive score;

Supplemental Figure 4

The clinical outcome of gastric cancer patients who received nivolumab monotherapy in the overall population, and its association with EBV or HER2 positivity. Kaplan–Meier estimates of progression-free survival (PFS, a) and overall survival (OS, b) of EBV-positive vs. negative patients. PSF (c), and OS (d) of HER2-positive vs. HER2-negative patients. EBV, Epstein-Barr virus; HER2, human epidermal growth factor receptor 2;

Supplemental Figure 5

(a-n) Kaplan–Meier estimates of progression-free survival (PFS) and overall survival (OS) of patients stratified by alteration of *TP53* (a, b), *CCNE1* (c,d), *ERBB2* (e,f), *PIK3CA* (g,h), *EGFR* (i, j), *CDK4* (k, l), *KRAS* (m,n). (o-p) Kaplan–Meier estimates of PFS (o) and OS (p) of patients with or without copy number alteration (CNA).

Supplemental Figure 6

The clinical outcome of MSI-H not detected gastric cancer patients who received nivolumab monotherapy, and its association with various cut-offs of bTMB. Kaplan–Meier estimates of progression-free survival (PFS, a) and overall survival (OS, b) of patients stratified by bTMB ≥4 vs. <4. PSF (c), and OS (d) of patients stratified by bTMB ≥6 vs. <6. bTMB, blood tumor mutation burden, MSI-H, microsatellite instability-high;

Supplemental Figure 7

The association between maxVAF dynamics and clinical outcome of gastric cancer patients treated with nivolumab monotherapy.
(a) Association between tumor response and changes of maxVAF between pre- vs. 6 weeks after the first dose (on-treatment) samples. (b) Correlation between maxVAF change from the baseline and degree of tumor shrinkage. (c) Comparison of DCR between cases with decreased ΔmaxVAF vs. non-decreased ΔmaxVAF. Kaplan–Meier estimates of progression-free survival (PFS, d) and overall survival (OS, e) of patients with decreased- vs. non-decreased-ΔmaxVAF. maxVAF; maximum variant allele frequency

Supplementary Table 1.

The association between tissue- and liquid- based biomarkers

(**a**) The concordance between HER2 status on tissue and ERBB2 CNA status on liquid.

(**b**) The association between PIK3CA alteration and EBV status.

(**c**) The concordance between MMR status and MSI status.

EBV, Epstein-Barr virus; HER2, human epidermal growth factor receptor 2; MMR, mismatch repair

**a)**		HER2 IHC/ISH status	
		Positive	Negative
	Amplification	1	0
ERBB2 CNA	Aneuploid	3	3
	No	1	23

**b)**	EBER-ISH	
	Positive	Negative
PIK3CA alteration (+)	2	4
PIK3CA alteration (-)	1	23

*p value: 0.0936

**c)**	MMR status		
	dMMR	pMMR	NA
MSI-H	3	0	0
MSI-H not detected	0	27	1

*MSI-dMMR concordance: 3/3 (100%)

Abbreviations: CNA, copy number alteration; MMR, mismatch repair-deficient; EBER-ISH, Epstein-Barr virus (EBV)-encoded RNA in-situ hybridization; ERBB2, epidermal growth factor receptor 2; HER2, human epidermal growth factor receptor 2; MSI-H, microsatellite instability-high; MMR, mismatch repair; NA, not available; PD-L1 CPS, programmed death ligand 1 combined positive score; *PIK3CA, phosphatidylinositol-4,5-bisphosphate 3-kinase catalytic subunit alpha;*

MMR, mismatch repair-proficient.

Supplemental Table 2. The comparison of the differences between platforms and schedules of the two studies that compared the utility of maxVAF changes and bTMB changes as an on-treatment biomarker

	Our study	Previous literature (ref. 46)
**Platforms**	Targeted NGS-based panel with 74 genes (Guardant 360, Guardant Health, Inc.)	Targeted NGS-based panel with 89 genes (Ambry Genetics)
**Schedules**		
Pre-treatment sample collection	Prior to 1st cycle	Prior to 1st cycle
Post-treatment sample collection	Prior to 2nd cycle	Prior to 3rd cycle

Supplemental Table 3. The gene composition of Guardant360

AKT1	CDK4	GNAQ	MLH1	RAF1
ALK	CDK6	GNAS	MPL	RB1
APC	CDKN2A	HNF1A	MTOR	RET
AR	CTNNB1	HRAS	MYC	RHEB
ARAF	DDR2	IDH1	NF1	RHOA
ARID1A	EGFR	IDH2	NFE2L2	RIT1
ATM	ERBB2	JAK2	NOTCH1	ROS1
BRAF	ESR1	JAK3	NPM1	SMAD4
BRCA1	EZH2	KIT	NRAS	SMO
BRCA2	FBXW7	KRAS	NTRK1	STK11
CCND1	FGFR1	MAP2K1	NTRK3	TERT
CCND2	FGFR2	MAP2K2	PDGFRA	TP53
CCNE1	FGFR3	MAPK1	PIK3CA	TSC1
CDH1	GATA3	MAPK3	PTEN	VHL
CDK12	GNA11	MET	PTPN11	

Supplemental Figure 1

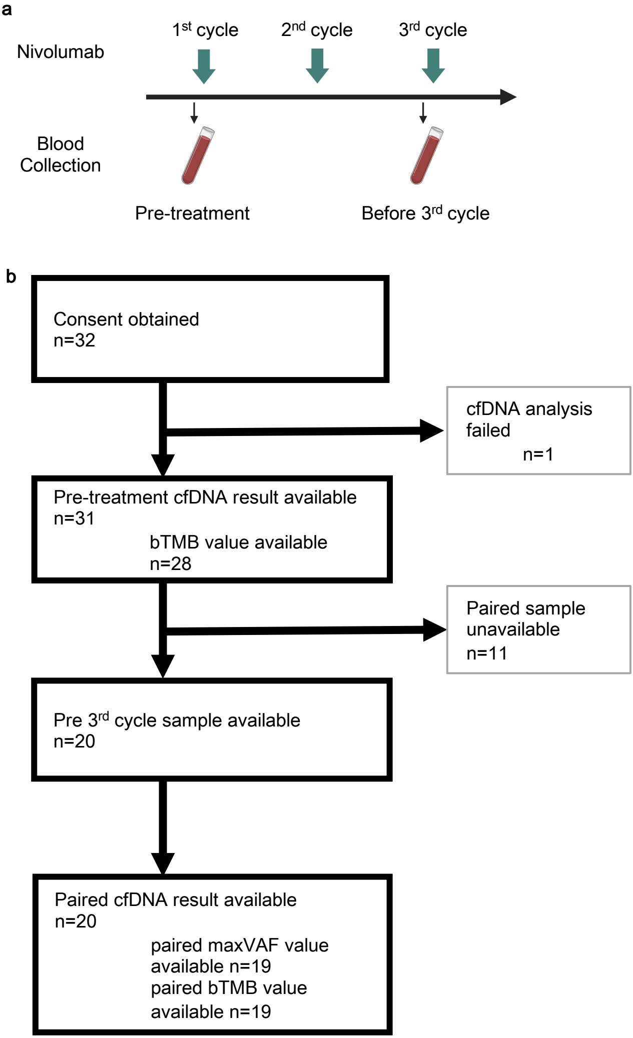

Supplemental Figure 2

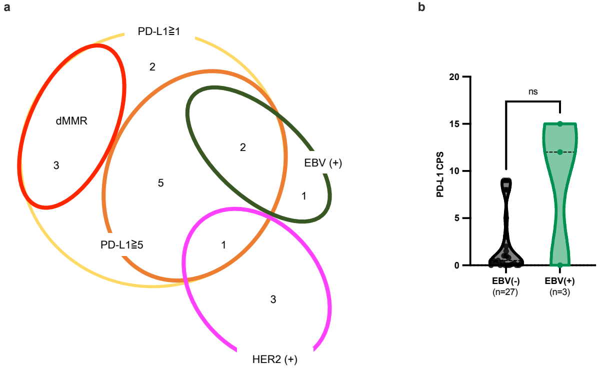

Supplemental Figure 3

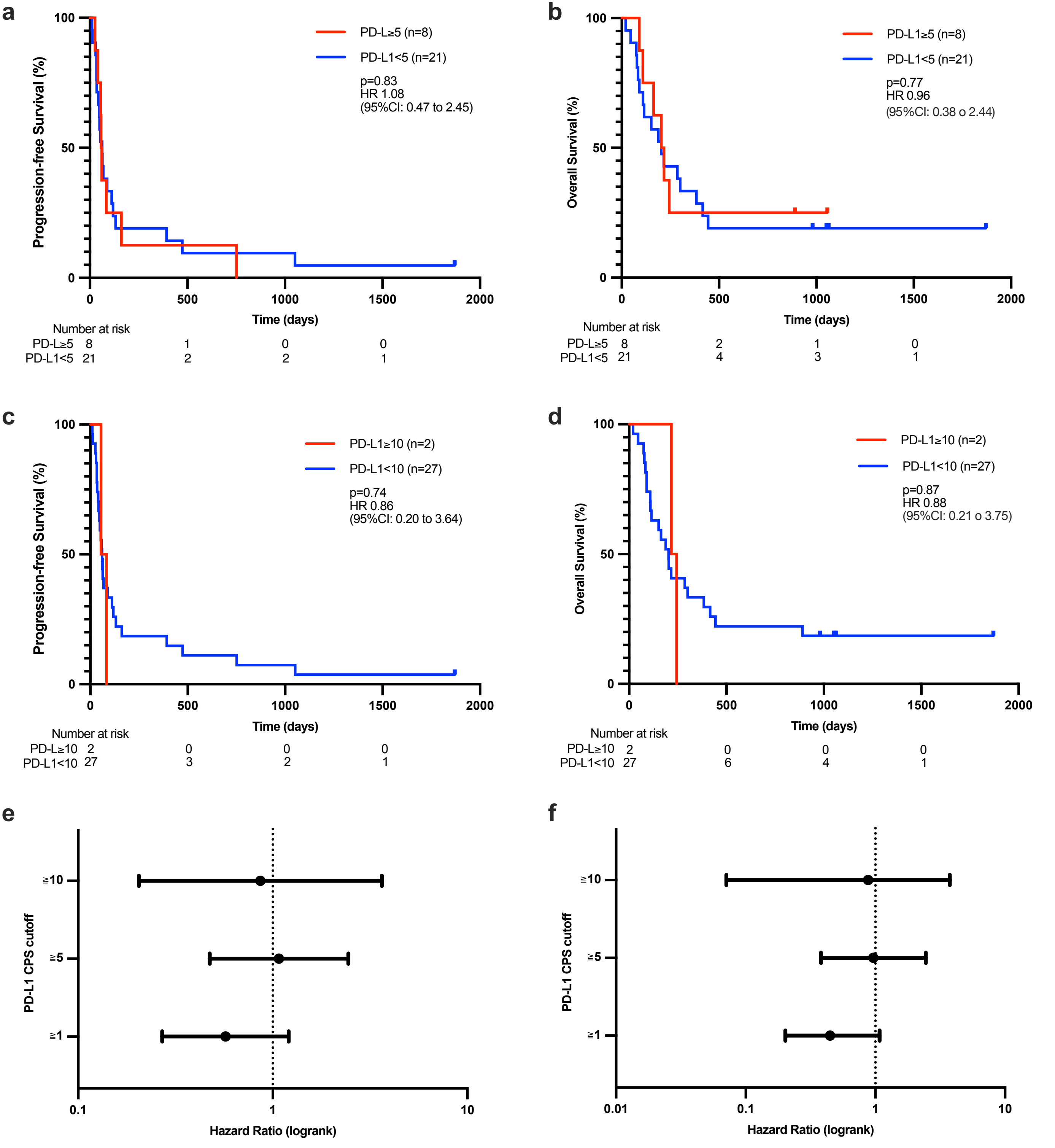

Supplemental Figure 4

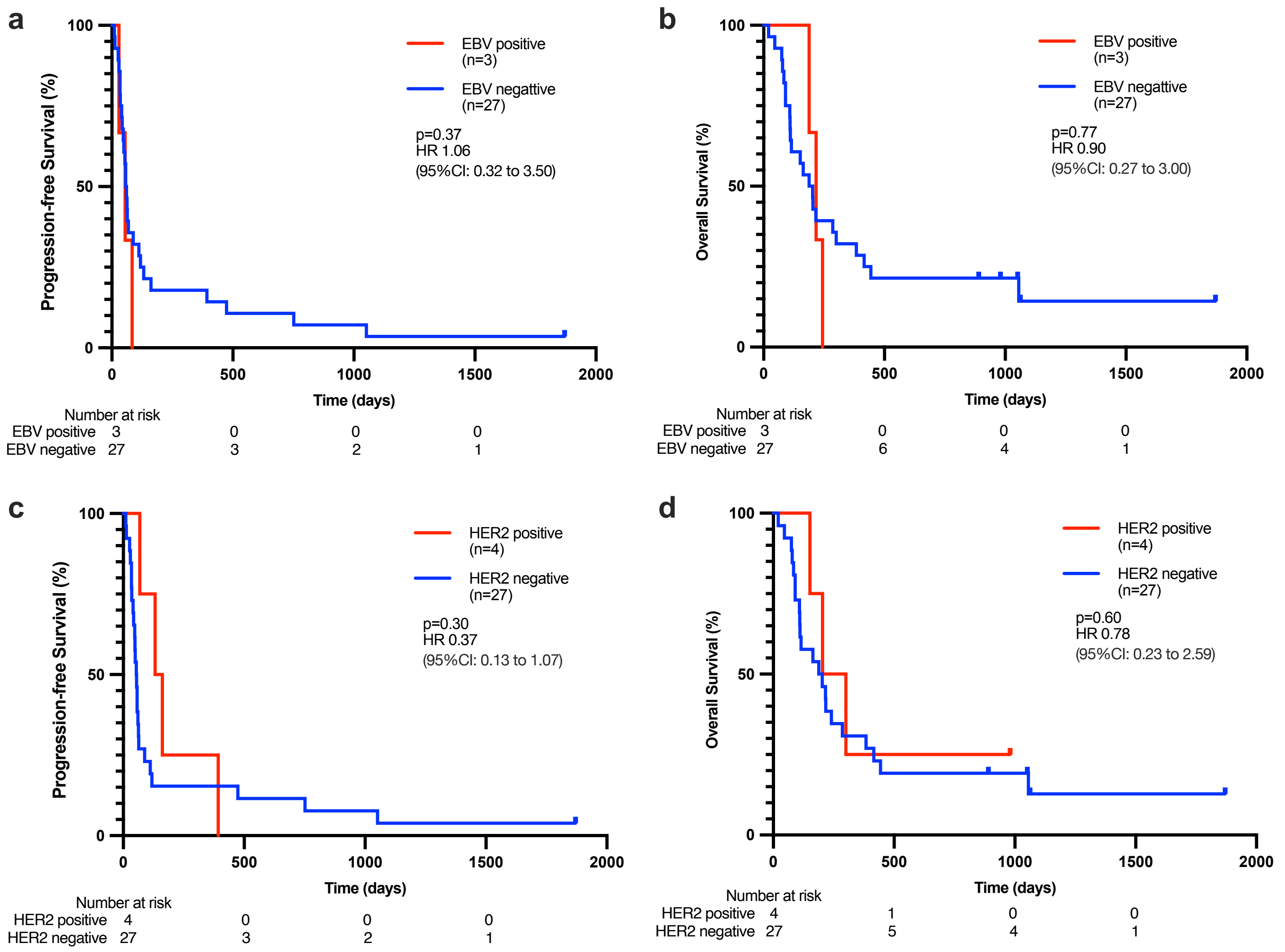

Supplemental Figure 5

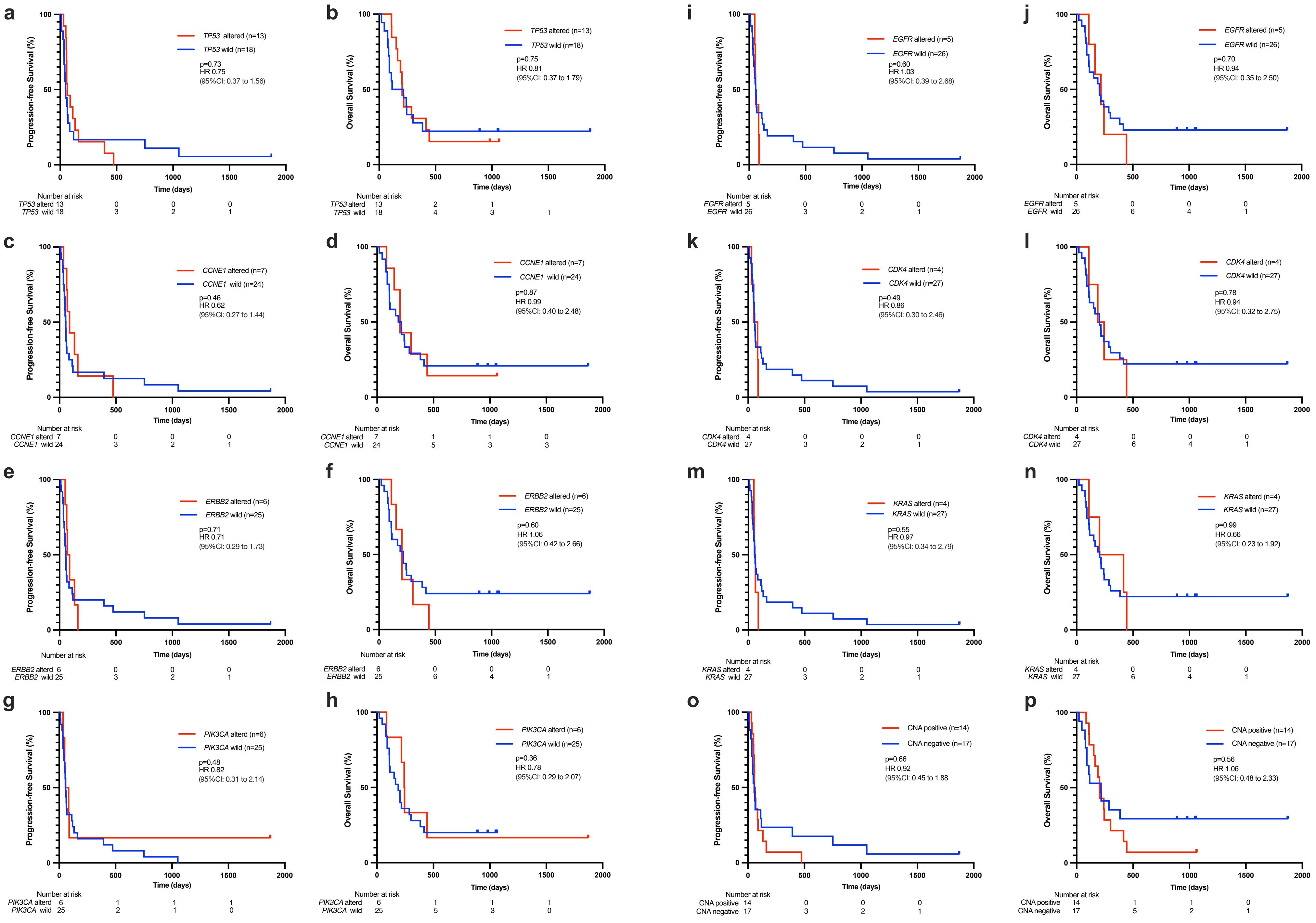

Supplemental Figure 6

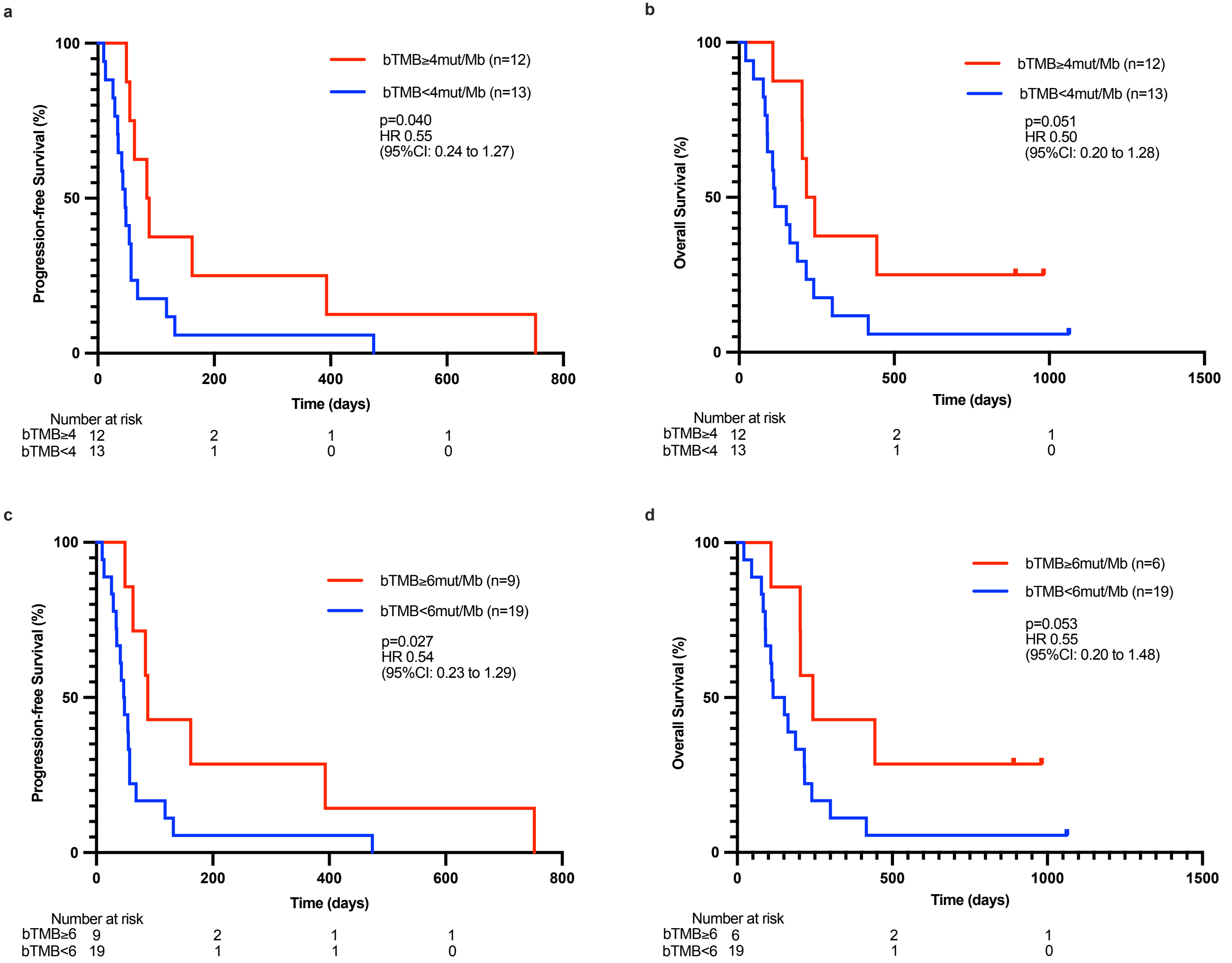

Supplemental Figure 7

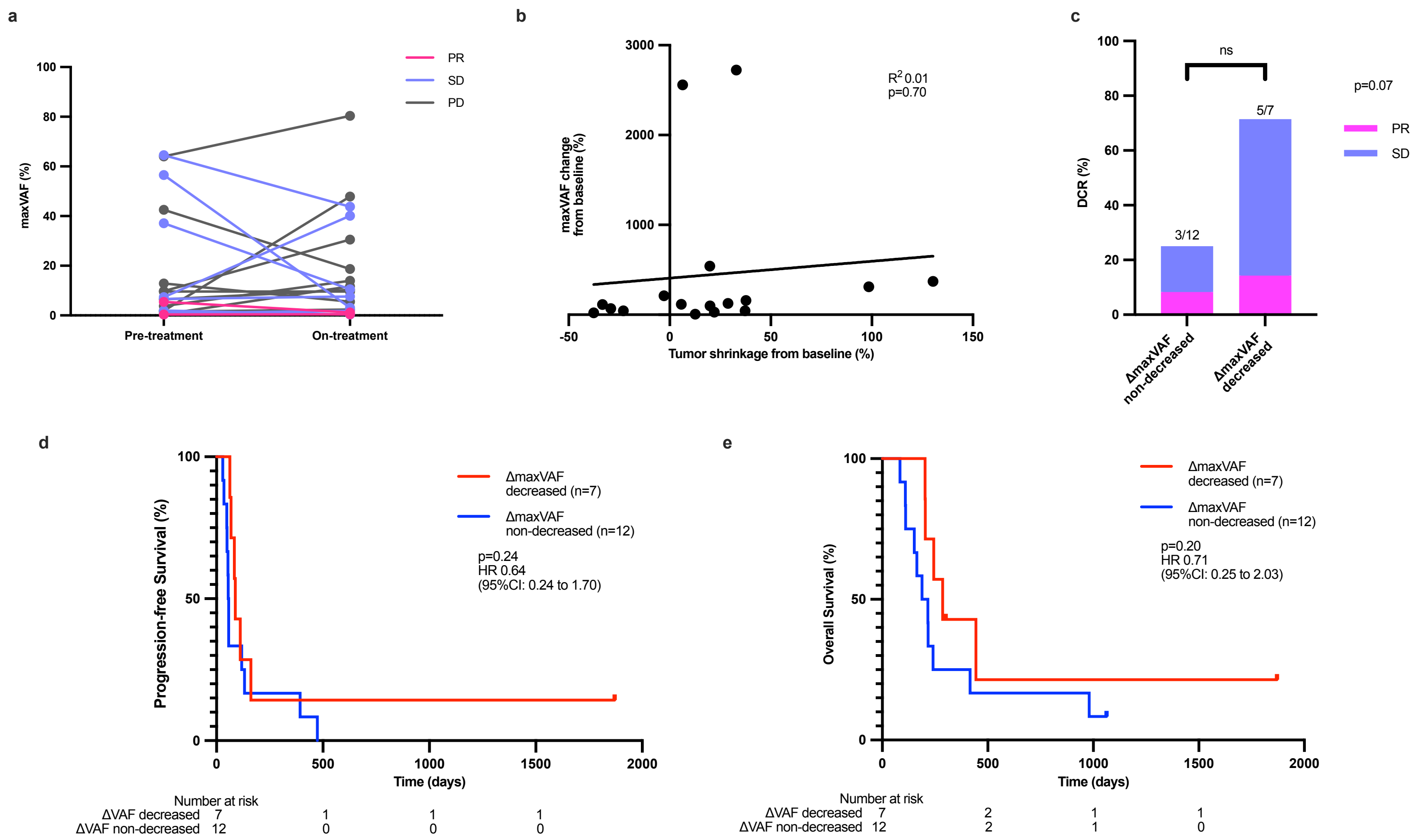

Supplement: Supplementary file 1 — Supplementary Information. [file 41598_2023_32645_MOESM1_ESM.pdf]
